# Addition of Financial Incentives to Mailed Outreach for Promoting Colorectal Cancer Screening: A Systematic Review and Meta-analysis

**DOI:** 10.1001/jamanetworkopen.2021.22581

**Published:** 2021-08-25

**Authors:** Antonio Facciorusso, Joshua Demb, Babu P. Mohan, Samir Gupta, Siddharth Singh

**Affiliations:** 1 Gastroenterology Unit, Ospedali Riuniti di Foggia, Foggia, Italy; 2 Moores Cancer Center, University of California at San Diego, La Jolla; 3 Division of Gastroenterology and Hepatology, University of Utah School of Medicine, Salt Lake City; 4 Section of Gastroenterology, Veterans Affairs San Diego Healthcare System, San Diego, California; 5 Division of Gastroenterology, University of California at San Diego, La Jolla

## Abstract

**Importance:**

Although screening decreases incidence of and mortality from colorectal cancer (CRC), screening rates are low. Health-promoting financial incentives may increase uptake of cancer screening.

**Objective:**

To evaluate the relative and absolute benefit associated with adding financial incentives to the uptake of CRC screening.

**Data Sources:**

PubMed, Cochrane Central Register of Controlled Trials, and Web of Science were searched from inception to July 31, 2020. Keywords and Medical Subject Headings terms were used to identify published studies on the topic. The search strategy identified 835 studies.

**Study Selection:**

Randomized clinical trials (RCTs) were selected that involved adults older than 50 years who were eligible for CRC screening, who received either various forms of financial incentives along with mailed outreach or no financial incentives but mailed outreach and reminders alone, and who reported screening completion by using recommended tests at different time points. Observational or nonrandomized studies and a few RCTs were excluded.

**Data Extraction and Synthesis:**

The review was reported in accordance with the Preferred Reporting Items for Systematic Reviews and Meta-analyses (PRISMA). Data were abstracted and risk of bias was assessed by 2 independent reviewers. Random-effects meta-analysis was conducted, heterogeneity was examined through subgroup analysis and metaregression, and quality of evidence was appraised.

**Main Outcomes and Measures:**

The primary outcome was CRC screening completion within 12 months of receiving the intervention.

**Results:**

A total of 8 RCTs that were conducted in the United States and reported between January 1, 2014, and December 31, 2020, were included. The trials involved 110 644 participants, of whom 53 444 (48.3%) were randomized to the intervention group (received financial incentives) and 57 200 (51.7%) were randomized to the control group (received no financial incentives). Participants were predominantly male, with 59 113 men (53.4%). Low-quality evidence (rated down for risk of bias and heterogeneity) suggested that adding financial incentives may be associated with a small benefit of increasing CRC screening vs no financial incentives (odds ratio [OR], 1.25; 95% CI, 1.05-1.49). With mailed outreach having a 30% estimated CRC screening completion rate, adding financial incentives may increase the rate to 33.5% (95% CI, 30.8%-36.2%). On metaregression, the magnitude of benefit decreased as the proportion of participants with low income and/or from racial/ethnic minority groups increased. No significant differences were observed by type of behavioral economic intervention (fixed amount: OR, 1.26 [95% CI, 1.05-1.52] vs lottery: OR, 1.06 [95% CI, 0.80-1.40]; *P* = .32), amount of incentive (≤$5: OR, 1.09 [95% CI, 1.01-1.18] vs >$5: OR, 1.25 [95% CI, 1.02-1.54]; *P* = .22), or screening modality (stool-based test: OR, 1.14 [95% CI, 0.92-1.41] vs colonoscopy: OR, 1.63 [95% CI, 1.01-2.64]; *P* = .18).

**Conclusions and Relevance:**

Adding financial incentives appeared to be associated with a small benefit of increasing CRC screening uptake, with marginal benefits in underserved populations with adverse social determinants of health. Alternative approaches to enhancing CRC screening uptake are warranted.

## Key Points

**Question:**

Does adding financial incentives to mailed outreach and reminders increase the rate of colorectal cancer screening?

**Findings:**

In this systematic review and meta-analysis of 8 randomized clinical trials with 110 644 participants, the addition of financial incentives to promotion interventions appeared to be associated with a modest benefit of increasing colorectal cancer screening completion compared with using no financial incentives. However, no clear benefit was observed in underserved populations with adverse social determinants of health.

**Meaning:**

Monetary incentives may not substantially increase colorectal cancer screening rates in populations with a traditionally low rate of screening completion.

## Teaser

This systematic review and meta-analysis evaluates whether use of monetary incentives encourage adults to undergo colorectal cancer screening.

## Introduction

Colorectal cancer (CRC) is the third most common cancer and the third-leading cause of cancer-related mortality in the world.^1^ Most cases of sporadic CRC arise from adenomas, following the well-recognized adenoma-carcinoma sequence.^2^ Systematic screening and surveillance programs have been implemented in several countries, and early detection of colon polyps with polypectomy has been associated with decreased CRC incidence and mortality.^3^

In the United States, the National Colorectal Cancer Roundtable set a goal of screening at least 80% of eligible adults for CRC by 2018^4^; however, national estimates for CRC screening completion have plateaued at approximately 68%.^5^ Several multicomponent strategies have been proposed and variably implemented to enhance CRC screening by increasing community demand (including through client reminders, client incentives, small and mass media promotions, and education), community access (including reducing structural barriers and out-of-pocket costs), and clinic or clinician participation in the delivery of screening services (including assessment and feedback, incentives, and/or reminders).^6^ Among community-directed interventions, outreach with mailed or in-person distribution of stool-based testing and patient navigation has been associated with significantly higher screening adherence, providing a consistent benefit across different patient populations.^7^ Meanwhile, patient education or reminders through telephone calls or letters has had a modest role in increasing screening completion.^7,8^

Financial incentives have been shown to promote healthy behavior, such as smoking cessation, vaccination, and regular physical activity, for a variety of conditions.^9^ Their association with improving cancer screening rates, including breast and cervical cancer screening, has been inconclusive.^10^ Studies that have evaluated the addition of financial incentives to mailed outreach and client reminders for CRC screening have reported variable success in different populations.^11,12,13^ Hence, we conducted a systematic review with meta-analysis of randomized clinical trials (RCTs) to evaluate the relative and absolute benefit associated with adding financial incentives to the uptake of CRC screening.

## Methods

We followed the Preferred Reporting Items for Systematic Reviews and Meta-analyses (PRISMA) reporting guideline and conducted the study according to a priori established protocol.^14^ We appraised the quality of evidence using the GRADE (Grading of Recommendations Assessment, Development and Evaluation) framework.

### Selection Criteria, Search Strategy, and Study Identification

Studies that were included in the meta-analysis were RCTs that met the following criteria: (1) patients were adults older than 50 years who were eligible for or not up-to-date with CRC screening (not up-to-date was defined as not having had a colonoscopy in the past 10 years, flexible sigmoidoscopy in the past 5 years, or stool testing in the past year) according to prevailing guidelines,^15,16^ (2) patients either received interventions, such as various forms of financial incentives (fixed or lottery, which may be unconditional or conditional on screening completion) in addition to CRC screening reminders and/or mailed outreach, or they were assigned to a comparator group that received no financial incentives but received CRC screening reminders and/or mailed outreach, and (3) patients reported outcomes, such as CRC screening completion, by using recommended tests at different time points up to 12 months after intervention. We excluded observational or nonrandomized studies and RCTs that compared different financial incentives without a usual care strategy,^17^ reported long-term data of previously published trials,^18^ or did not report specific CRC screening modalities.^19^

We searched PubMed, Cochrane Central Register of Controlled Trials, and Web of Science from inception to July 31, 2020, using keywords and Medical Subject Headings terms, including *financial incentives*, *motivation*, and *behavioral economics* in combination with *cancer screening* and *colonoscopy*. Two of us (A.F. and S.S.) independently reviewed the titles and abstracts of the studies that were identified in the search to exclude studies that did not address the research question of interest according to prespecified inclusion and exclusion criteria. Conflicts in study selection at this stage were resolved by consensus, whereby we referred back to the original article in consultation with another investigator (S.G.). We conducted a manual search of abstracts from major gastroenterology conferences that were held between 2015 and 2020 to identify additional abstracts on the topic.

### Data Abstraction, Risk-of-Bias Assessment, and Outcomes

Two of us (A.F. and S.S.) abstracted study-, patient-, and treatment-related characteristics onto a standardized form. Discrepancies were resolved by reviewing the articles jointly and, in case of a disagreement, in consultation with another investigator (S.G.). We assessed the risk of bias of individual studies in the context of the primary outcome, using the Joanna Briggs Institute critical appraisal tool.^20^ This tool was preferred over the Cochrane risk of bias tool because it allows for a more detailed assessment of health policy interventions.

The primary outcome was completion of CRC screening within 12 months of receiving the intervention. To evaluate the stability of association and identify potential sources of heterogeneity, we performed preplanned subgroup analyses on the basis of the (1) type of behavioral economic intervention or incentive: fixed amount that was conditional on screening completion vs lottery; (2) amount of incentive: $5 or less vs more than $5, including lottery incentive; (3) screening modality: stool-based test (eg, fecal occult blood test [FOBT] or fecal immunochemical test [FIT]) vs colonoscopy; (4) baseline outreach modality: mailed outreach with a stool testing kit vs mailed or electronic reminders only; (5) time frame of outcome assessment: within 3 months vs more than 3 months; and (6) risk of bias: low to moderate vs high.

To evaluate sources of heterogeneity, we performed a metaregression that accounted for study-level variables, including sex (proportion of male participants), race/ethnicity (proportion of participants from racial/ethnic minority groups), and mean annual household income for the zip code of residence. We also conducted sensitivity analyses of studies in which only the individuals who were not up-to-date on CRC screening were randomized.

### Statistical Analysis

We used DerSimonian and Laird random-effects model to estimate odds ratios (ORs) and 95% CIs for CRC screening completion in individuals who received financial incentives (intervention group) vs those who received no financial incentives (control group).^21^ Post hoc sensitivity analysis using the Hartung-Knapp-Sidik-Jonkman method was also performed. Statistical heterogeneity was assessed using the *I*^2^ statistic, with *I*^2 ^≥50% considered substantial heterogeneity.^22^ Small-study effects (ie, publication bias) were assessed visually with funnel plots and statistically with an Egger regression test.^23^

All statistical analyses were conducted using RevMan, version 5 (Cochrane Collaboration) and *metafor* package in R, version 3.0.2 (R Foundation for Statistical Computing).^24^ For all other calculations, a 2-tailed *P* < .05 was considered statistically significant.

The GRADE approach was used to rate certainty in summary effect estimates.^25^ In this approach, direct evidence from RCTs starts at high quality and can be rated down to moderate, low, or very low quality depending on the risk of bias in the body of evidence, indirectness, imprecision, inconsistency (or heterogeneity), and/or publication bias. For imprecision, evidence was rated down even if the 95% CI did not cross unity if the optimal information size (measure of fragility) was smaller than 200 events of CRC screening across all studies.^26^

To estimate the magnitude of incremental benefit associated with financial incentives, we generated estimates of absolute event rates (or absolute risk) by calculating the estimated risk difference derived directly from individual studies or by transforming the OR into the risk difference. For this analysis, we estimated the pooled mailed outreach response rate of 30% that was observed across included RCTs as the assumed control risk; mailed outreach was the most commonly used intervention across trials. The risk difference, which represented the difference in event rates between the intervention group and control group, was added back to the assumed control risk to generate an estimate of the absolute risk for each intervention. The 95% CIs for the estimates were generated from the 95% CIs of the ORs.

## Results

**Figure 1.**
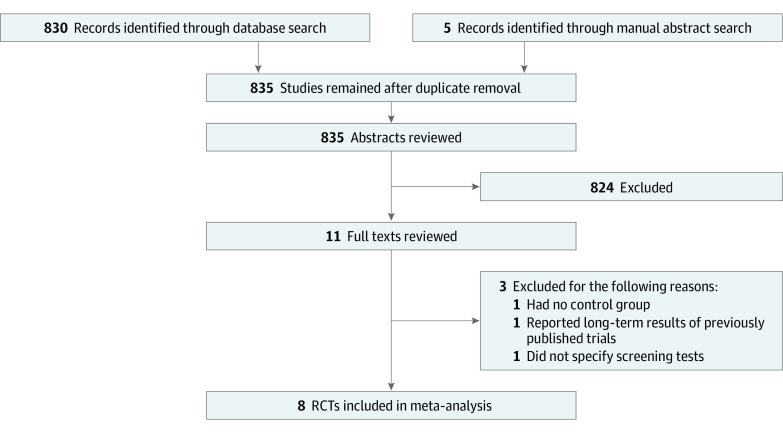
Study Selection Flowsheet RCT indicates randomized clinical trial.

The search strategy identified 835 studies, of which 8 RCTs met the inclusion criteria and were included in the meta-analysis (Figure 1).^11,12,13,27,28,29,30,31^ Table 1 summarizes the characteristics of the 8 RCTs. Overall, these trials involved 110 644 participants, of whom 53 444 (48.3%) were randomized to the intervention group and offered various financial incentives and 57 200 (51.7%) were randomized to the control group and offered no financial incentives. All 8 RCTs were conducted in the United States and were published between January 1, 2014, and December 31, 2020.

**Table 1.** Characteristics of Included Randomized Clinical Trials Comparing Different Financial Incentives for Increasing Colorectal Cancer Screening Uptake in the US

Source	Period	Follow-up	Intervention; No. of individuals; source of incentives	Control; No. of individuals	Annual household income^a^	Primary outcome, pooled rates in each group (95% CI)
Green et al,^27^ 2019	2017-2018	6 mo	Group 1: $10 conditional incentive + mailings; n = 270; NR Group 2: lottery with conditional 1:10 chances of winning $50 + mailings; n = 284; NR	First mailing with screening information, second mailing with FIT kit, and eventual third reminder mailing; n = 284	<$50 000, No. (%) Group 1: 91 (36%) Group 2: 102 (40%) Control group: 99 (37.6%)	Completion of any CRC screening: FIT and colonoscopy Group 1: 76.7% (71.6%-81.7%) Group 2: 74.6% (69.6%-79.7%) Control group: 71.5% (66.2%-76.7%)
Gupta et al,^11^ 2016	2013-2016	12 mo	Group 1: $5 conditional incentive + mailings; n = 1000; local health system Group 2: $10 conditional incentive + mailings; n = 1000; local health system	Mailing with FIT kit, 2 automated telephone reminders, up to 2 eventual live telephone reminders within 4 weeks; n = 6565	Neighborhood poverty rate: >30%, No. (%) Group 1: 302 (30.2%) Group 2: 314 (31.4%) Control group: 2002 (30.5%)	Completion of FIT Group 1: 39.2% (36.2%-42.2%) Group 2: 34.6% (31.7%-37.5%) Control group: 36.2% (35.1%-37.4%)
Kullgren et al,^12^ 2014; stage 1	2012	1 mo	Group 1: $5 conditional incentive + mailing; n = 158; local health system Group 2: $10 conditional incentive + mailing; n = 185; local health system Group 3: $20 conditional incentive + mailing; n = 201; local health system	Mailing with FOBT kit only; n = 167	Mean Group 1: $43 346 Group 2: $42 945 Group 3: $39 296 Control group: $40 869	Completion of FOBT Group 1: 39.2% (31.6%-46.9%) Group 2: 44.9% (37.7%-52%) Group 3: 46.3% (39.4%-53.2%) Control group: 38.3% (30.9%-45.7%)
Kullgren et al,^12^ 2014; stage 2	2012	1 mo	Group 1: $5 conditional incentive + mailing; n = 213; local health system Group 2: lottery with conditional 1:10 chances of winning $50 + mailing; n = 209; local health system Group 3: conditional $500 raffle + mailing; n = 176; local health system	Mailing with FOBT kit only; n = 238	Mean Group 1: $41 524 Group 2: $41 853 Group 3: $40 789 Control group: $40 857	Completion of FOBT Group 1: 37.6% (31.1%-44.1%) Group 2: 49.3% (42.5%-56.1%) Group 3: 40.3% (33.1%-47.6%) Control group: 29.4% (23.6%-35.2%)
Mehta et al,^30^ 2017	2016	3 mo	Group 1: email with telephone number to schedule colonoscopy; n = 748; employer Group 2: email with active choice to opt into or opt out of scheduling (active choice) + conditional $100 incentive; n = 748; employer	Email with active choice only; n = 749	Mean Group 1: $66 412 Group 2: $66 553 Control group: $67 787	Completion of colonoscopy Group 1: 1.5% (0.6%-2.3%) Group 2: 3.7% (2.4%-5.1%) Control group: 1.6% (0.7%-2.5%)
Mehta et al,^28^ 2019	2015-2018	6 mo	Group 1: $10 unconditional incentive + mailing; n = 224; NR Group 2: $10 conditional incentive + mailing; n = 224; NR Group 3: lottery with conditional 1:10 chances of winning $100 + mailing; n = 226; NR	Mailing with FIT kit only; n = 223	Mean Group 1: $30 797 Group 2: $30 797 Group 3: $31 113 Control group: $30 797	Completion of FIT Group 1: 31.7% (25.6%-37.8%) Group 2: 26.8% (21%-32.6%) Group 3: 24.3% (18.7%-29.9%) Control group: 32.7% (26.6%-38.9%)
Mehta et al,^13^ 2020	2017	3 mo	$10 conditional incentive for web-based risk assessment + $25 unconditional incentive for colonoscopy completion; n = 990; employer	Web-based risk assessment and direct access to colonoscopy scheduling; n = 987	Median (IQR) Intervention group: $73 231 ($47 287-$94 920) Control group: $73 231 ($47 287-$94 395)	Completion of colonoscopy Group 1: 31.9% (29%-34.8%) Control group: 19.5% (17%-21.9%)
Mehta et al,^29^ 2020	2017	3 mo	Opt-in messaging + conditional lottery with 1:5 chances of winning $100; n = 141; NR	Opt-in text messaging to receive FIT + eventual 3 reminders; n = 140	Median (IQR) Intervention group: $29 972 ($29 972-$30 797) Control group: $29 972 ($29 972-$30 797)	Completion of FIT Group 1: 12.1% (6.7%-17.4%) Control group: 12.1% (6.7%-17.6%)
Slater et al,^31^ 2018	2014-2015	3 mo	$20 conditional incentive + mailings; n = 47 195; NR	3 mailings with a number to obtain patient navigation on the same schedule as intervention but 15 mo later; n = 47 099	Mean income, (% of participants classified as having low income) Intervention group: $29 967 (63.5%) Control group: $29 780 (63.2%)	Completion of colonoscopy Absolute events not reported.

Abbreviations: CRC, colorectal cancer; FIT, fecal immunochemical test; FOBT, fecal occult blood test; IQR, interquartile range; NR, not reported.

^a^ Mean household income for zip code of residence.

The choice of screening test varied between studies, including FOBT (1 RCT)^12^ or FIT (3 RCTs),^11,28,29^ colonoscopy (3 RCTs),^13,30,31^ and any approved CRC screening test from a menu of options (1 RCT).^27^ Trials used various behavioral economic interventions, including small amounts of fixed incentives, ranging from $5 to $20 (except in 1 study in which the incentive was $100), that were typically conditional on completion of a screening test (except in 1 trial with unconditional fixed incentive^28^) or a lottery that presented 1:10 chances of earning larger amounts, ranging from $50 to $100, for test completion (1 trial offered a chance to enter a raffle with a $500 prize^12^). For all trials, the control group consisted of different degrees of outreach or reminders, such as mailing with FOBT or FIT kit plus reminders in 4 RCTs,^11,12,27,28^ a web-based algorithm with direct access to colonoscopy scheduling in 1 RCT,^13^ an active choice approach in 2 RCTs,^29,30^ and patient navigation in 1 RCT.^31^

The outcome (completion of CRC screening) was assessed at 1 month in 1 RCT,^12^ 3 months in 4 RCTs,^13,29,30,31^ 6 months in 2 RCTs,^27,28^ and 12 months in 1 RCT.^11^ Main baseline demographic characteristics of study participants are reported in eTable 1 in the Supplement. Overall, the participants were composed of 59 113 men (53.4%) and 51 531 women (46.6%). All of the included RCTs were well balanced in terms of baseline variables. Traditionally underrepresented racial/ethnic minority groups were prevalent (ie, comprised >50% of participants) in 4 RCTs.^11,28,29,30^ A low educational status (high school diploma or less) was registered in more than 80% of participants in 2 RCTs.^12,31^ Two trials included individuals who could have had an up-to-date status on CRC screening.^13,30^

Risk-of-bias assessment was performed in the context of the primary outcome. Given the nature of the intervention, participants could not be blinded to the treatment randomization. Three studies were deemed to be moderate quality,^11,27,28^ and the other RCTs^12,13,29,30,31^ were rated as low quality because of inadequate follow-up, lack of physician blinding, or mixed population (individuals with not up-to-date and up-to-date CRC screening status) (eTable 2 in the Supplement).

### Completion of CRC Screening

**Figure 2.**
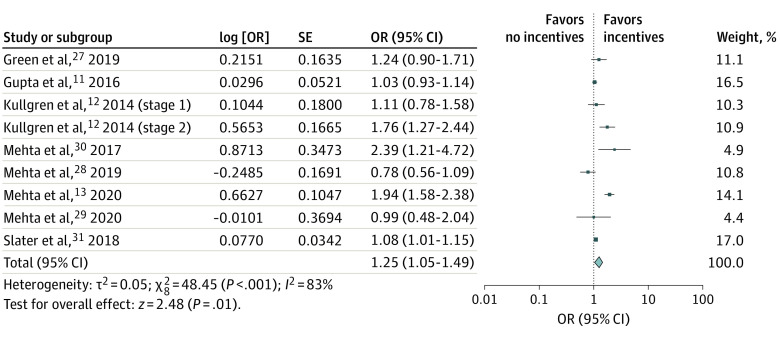
Forest Plot of the Association Between Adding Financial Incentives and Increasing Colorectal Cancer Screening Uptake OR indicates odds ratio.

Based on meta-analysis, adding financial incentives was associated with 25% higher odds of CRC screening completion in the intervention group vs control group (OR, 1.25; 95% CI, 1.05-1.49), although considerable heterogeneity was observed (*I*^2^ = 83%) (Figure 2). Post hoc sensitivity analysis using the Hartung-Knapp-Sidik-Jonkman method yielded similar estimates (OR, 1.26; 95% CI, 0.97-1.64).

To examine the stability of association and identify potential sources of heterogeneity, we performed multiple subgroup analyses (Table 2). Overall, no significant differences were observed in the magnitude of benefit by type of financial incentive (fixed: OR, 1.26 [95% CI, 1.05-1.52] vs lottery: OR, 1.06 [95% CI, 0.80-1.40]; *P* for interaction = .32), amount of incentive (≤$5: OR, 1.09 [95% CI, 1.01-1.18] vs >$5: OR, 1.25 [95% CI, 1.02-1.54]; *P* = .22), screening modality (FOBT or FIT: OR, 1.14 [95% CI, 0.92-1.41] vs colonoscopy: OR, 1.63 [95% CI, 1.01-2.64]; *P* = .18), and baseline outreach modality (mailed outreach with FOBT or FIT kit: OR, 1.13 [95% CI, 0.91-1.41] vs mailed or electronic reminders only: OR, 1.48 [95% CI, 0.95-2.30]; *P* = .28). The benefit associated with adding financial incentives was higher in studies in which screening completion was assessed within 3 months rather than longer than 3 months (OR, 1.45 [95% CI, 1.06-1.98] vs OR, 1.01 [95% CI, 0.83-1.23]; *P* = .03). In addition, significant differences were observed in the meta-analysis of risk of bias, with 3 trials^11,27,28^ that were at low to moderate risk of bias showing no significant benefit vs 5 trials^12,13,29,30,31^ that were at high risk of bias (OR, 1.01 [95% CI, 0.87-1.18] vs OR, 1.51 [95% CI, 1.08-2.12]; *P* = .03) (Figure 2).

**Table 2.** Subgroup Comparisons of Financial Incentives Tested in the Included Randomized Clinical Trials

Variable	Subgroup	No. of studies	No. of individuals	OR (95% CI)	Within-group heterogeneity (*I*^2^), %	*P* value for difference between groups
Type of incentive	Fixed vs control	8	Fixed: 52 408 Control: 56 312	1.26 (1.05-1.52)	86	.32
	Lottery vs control	4	Lottery: 1036 Control: 885	1.06 (0.80-1.40)	80	
Amount of the financial incentive	≤$5 vs control	3	$5: 1371 Control: 6970	1.09 (1.01-1.18)	0	.22
	>$5 vs control	7	>$5: 51 037 Control: 56 074	1.25 (1.02-1.54)	87	
Screening test	FOBT or FIT	6	Incentives: 5134 Control: 6150	1.14 (0.92-1.41)	67	.18
	Colonoscopy	4	Incentives: 48 814 Control: 49 123	1.63 (1.01-2.64)	93	
Baseline outreach modality	Mailed outreach with FOBT or FIT kit	4	Incentives: 4370 Control: 7477	1.13 (0.91-1.41)	71	.28
	Mailed or electronic reminders only	5	Incentives: 49 822 Control: 48 975	1.48 (0.95-2.30)	91	
Timing of assessment	3 mo	6	Incentives: 51 037 Control: 56 074	1.45 (1.06-1.98)	87	.03
	>3 mo	3	Incentives:1245 Control: 6135	1.01 (0.83-1.23)	50	
Study quality	Low to moderate risk of bias	3	Incentives: 3369 Control: 7212	1.01 (0.87-1.18)	25	.03
	High risk of bias	5	Incentives: 48 409 Control: 48 309	1.51 (1.08-2.12)	90	

Abbreviations: FIT, fecal immunochemical test; FOBT, fecal occult blood test; OR, odds ratio.

**Figure 3.**
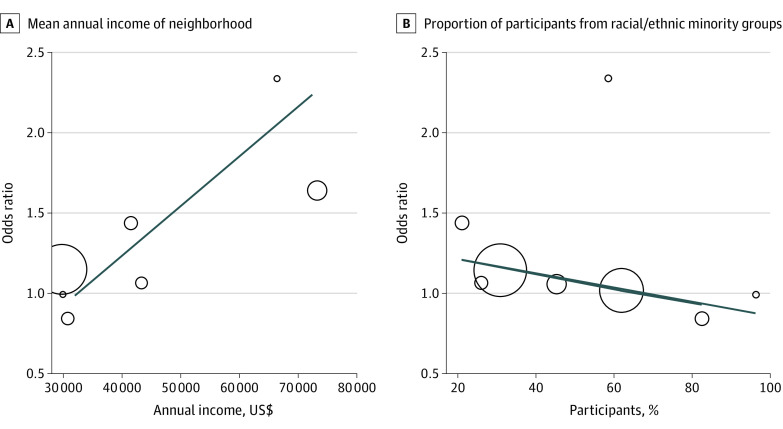
Metaregression of the Association of Financial Incentives With Neighborhood Income Level and Participants From Racial/Ethnic Minority Groups Each circle corresponds to a single study, and the sizes of the circles are proportional to the precision of each study. The estimated regression line indicates the correlation between the variables.

On metaregression, the magnitude of benefit associated with adding financial incentives decreased as the proportion of trial participants with low income and from racial/ethnic minority groups increased (Figure 3). This finding was able to explain the heterogeneity observed in overall estimates; sex distribution of the cohorts had no implication for the effect estimate.

On sensitivity analysis, when focusing only on trials that included participants with a not up-to-date CRC screening status, the result of adding financial incentives was not statistically significant (7 RCTs; OR, 1.10; 95% CI, 0.97-1.24). Because of the small number of studies, we opted against formal assessment for publication bias.

### Quality of Evidence

The overall body of evidence^11,12,13,27,28,29,30,31^ that supported adding financial incentives vs reminders only for improving CRC screening uptake rates was rated as low quality, with downgrading for serious risk of bias and considerable heterogeneity. On examining the overall magnitude of benefit, with a pooled estimated CRC screening rate of 30% (95% CI, 14.4%-45.8%) with mailed outreach of stool-based screening tests, adding financial incentives may modestly increase the CRC screening rate to 33.5% (95% CI, 30.8%-36.2%).

## Discussion

Despite evidence of the advantages of CRC screening, it remains underused in the United States, particularly in low-income populations and among individuals from minority racial/ethnic groups.^32^ Several population health interventions that focus on increasing demand through reminders and access through mailed outreach with stool-based tests have been shown to improve screening. In this systematic review and meta-analysis of 8 RCTs, we examined the results of adding financial incentives to reminders and/or mailed outreach (compared with reminders only) as a population-level intervention to enhance screening uptake.

Several key observations can be made regarding the results. First, financial incentives in addition to other interventions, such as mailed outreach or reminders, may be associated with a small incremental benefit for CRC screening uptake. With mailed outreach having an estimated benefit of 30% CRC screening completion, adding financial incentives may increase the completion rate to 33.5%. The overall body of evidence supporting this finding was low quality because of serious risk of bias in included studies and considerable heterogeneity. This treatment effect was smaller when restricted to higher-quality studies.

Second, the magnitude of benefit was similar regardless of the incentive framework (unconditional or conditional and fixed amount vs lottery), the amount of incentive (≤$5 vs >$5), screening test of interest, and baseline outreach modality. Financial incentives were more advantageous when screening completion was evaluated within 3 months of intervention.

Third, the incremental benefit associated with adding financial incentives was inversely associated with the application of the intervention to traditionally underserved populations; that is, the magnitude of benefit was lower in RCTs that were conducted in low-income neighborhoods and communities of racial/ethnic minority groups. Furthermore, on sensitivity analysis of only the trials in which all individuals were not up-to-date with CRC screening, no clear benefit associated with adding financial incentives was observed.

Overall, these findings suggest that, although adding financial incentives may be associated with a modest benefit of improving CRC screening, especially in the short term (probably nudging those who were inclined to undergo screening), financial incentivization may not address the concerns related to low screening uptake in underserved populations with a high prevalence of adverse social determinants of health.

Successful strategies to increase CRC screening completion often are multimodal and need to be tailored to populations of interest. In the present review, all patients received some reminders or mailed outreach for CRC screening. Patients also were randomized to receive either financial incentives or no financial incentives. In this setting, we observed a modest benefit with financially incentivizing screening completion. Testing various behavioral economic theories of loss-aversion or gain-framed incentives or different amounts of incentives did not significantly change the magnitude of benefit, particularly in individuals who were not up-to-date with screening. Monetary incentive was generally less than $20 when fixed incentives were offered. Larger incentive amounts may be perceived as coercion and may be untenable within the framework of population-level programmatic screening.

The observation of higher screening completion rates when examined within 3 months of intervention, but not at longer periods, was consistent with incentivization theories of behavioral economics: that is, offering incentives may nudge people who are inclined to complete CRC screening, which may lead to short-term change, but also has the potential to crowd out intrinsic motivations, which may lead individuals to regress to baseline behaviors in the long term.^33^ This observation was also confirmed in a long-term outcome analysis in 1 trial.^18^

Contrary to expectations, we found a lower magnitude of benefit associated with financial incentives among participants from racial/ethnic minority groups and low-income populations. This result appeared to be in contrast with the observation in the trial by Green et al^27^ that financial incentives may reduce health disparities, albeit only marginally, in this setting. Financial incentives represent a nudge that makes desired activities easier to do. The results suggest that for individuals with higher income and favorable social determinants, financial incentives, particularly those centered on loss aversion, may serve this purpose and tip the balance toward screening completion. On the other hand, underserved individuals may have such low awareness as well as face limited access and systemic barriers to health promotion that small incentive amounts may not be sufficient to overcome the systemic challenges. Other factors, such as health literacy regarding how the incentive was presented, could create higher barriers to perceiving the benefit, in particular when conditional or lottery-based incentives were offered.

### Limitations

This study has several limitations. First, considerable heterogeneity was observed, which could be partly explained through subgroup analyses and metaregression. However, because of the small number of trials, we were unable to perform multivariable metaregression or detailed analyses of the various behavioral economic incentives. The risk of both type I and type II errors was high in the metaregression and sensitivity analysis; thus we call for caution when interpreting these results. Moreover, relevant baseline characteristics of the study populations were missing in some of the included trials.

Second, considerable differences were observed in the baseline risk of screening completion in the control group. Hence, to contextualize the magnitude of benefit associated with adding financial incentives to mailed outreach, we used a pooled mailed outreach response rate of 30% across trials to estimate absolute benefit. Moreover, although the definition of the not up-to-date status was homogeneous in the included trials, information on when previous CRC screening tests were performed was lacking, which might represent a bias in the interpretation of the results.

Third, given the lack of patient-level data, inferences about the implication of certain variables for individuals were deduced from inferences about the groups to which those individuals belonged, thus increasing the risk for ecological bias.^34^ Fourth, we did not examine the cost-effectiveness of adding financial incentives to enhance CRC screening interventions.

## Conclusions

In this study, the addition of financial incentives to mailed outreach or reminder systems appeared to be associated with a small benefit of increasing CRC screening completion. However, this result may not be significant in underserved communities with traditionally low CRC screening completion. Alternative strategies to enhance CRC screening uptake in underserved populations as well as community engagement are warranted.

## Notes

Supplement. **eTable 1.** Demographical and Clinical Characteristics of Individuals Enrolled in the Included RCTs Comparing Different Financial Incentives to Increase CRC Screening Uptake **eTable 2.** Risk of Bias Assessment in Individual RCTs of Financial Incentives, Using the Joanna Briggs Institute Critical Appraisal Tool
