# Supplementary material for: Addition of Financial Incentives to Mailed Outreach for Promoting Colorectal Cancer Screening: A Systematic Review and Meta-analysis
Source: JAMA Netw Open. 2021 Aug 25;4(8):e2122581. doi: 10.1001/jamanetworkopen.2021.22581 (PMC8387849; doi:10.1001/jamanetworkopen.2021.22581)
Supplement: Supplement. — eTable 1. Demographical and Clinical Characteristics of Individuals Enrolled in the Included RCTs Comparing Different Financial Incentives to Increase CRC Screening Uptake eTable 2. Risk of Bias Assessment in Individual RCTs of Financial Incentives, Using the Joanna Briggs Institute Critical Appraisal Tool [file jamanetwopen-e2122581-s001.pdf]

Supplementary Online Content

Facciorusso A, Demb J, Mohan BP, Gupta S, Singh S. Addition of financial incentives to mailed outreach for promoting colorectal cancer screening: a systematic review and meta-analysis. *JAMA Netw Open*. 2021;4(8):e2122581. doi:10.1001/jamanetworkopen.2021.22581

eTable 1. Demographical and Clinical Characteristics of Individuals Enrolled in the Included RCTs Comparing Different Financial Incentives to Increase CRC Screening Uptake

eTable 2. Risk of Bias Assessment in Individual RCTs of Financial Incentives, Using the Joanna Briggs Institute Critical Appraisal Tool

This supplementary material has been provided by the authors to give readers additional information about their work.

© 2021 Facciorusso A et al. *JAMA Network Open.*

eTable 1. Demographical and Clinical Characteristics of Individuals Enrolled in the Included Randomized Controlled Trials Comparing Different Financial Incentives to Increase CRC Screening Uptake

Study, Year	Age	Gender male	Race non-white	Low education (high school or less)	MedicAid Insurance	Not up-to-date for CRC screening
Green, 2019[27]	>60 years<br>Arm 1: 123 (45.6%)<br><br>Arm 2: 143 (50.4%)<br><br>Control: 137 (48.2%)	Arm 1: 90 (33.3%)<br><br>Arm 2: 96 (33.5%)<br><br>Control: 107 (37.7%)	Arm 1: 124 (45.3%)<br><br>Arm 2: 136 (47.1%)<br><br>Control: 145 (50.9%)	Arm 1: 37 (14.1%)<br><br>Arm 2: 57 (20.8%)<br><br>Control: 41 (14.9%)	Arm 1: 22 (8.1%)<br><br>Arm 2: 23 (8.1%)<br><br>Control: 14 (4.9%)	100%
Gupta, 2016[11]	Arm 1: 56 (53-60)<br><br>Arm 2: 56 (53-60)<br><br>Control: 56 (53-60)	Arm 1: 390 (39%)<br><br>Arm 2: 356 (35.6%)<br><br>Control: 2523 (38.4%)	Arm 1: 619 (61.9%)<br><br>Arm 2: 632 (63.2%)<br><br>Control: 4137 (63%)	NR	NR	100%
Kullgren, 2014[12]<br><br>Stage 1	Arm 1: 62.7±7.2<br><br>Arm 2: 61.6±7.1<br><br>Arm 3: 60.7±6.8<br><br>Control: 61.9±7.6	Arm 1: 157 (99%)<br><br>Arm 2: 185 (100%)<br><br>Arm 3: 201 (99%)<br><br>Control: 167 (100%)	Arm 1: 40 (26%)<br><br>Arm 2: 41 (22%)<br><br>Arm 3: 53 (26%)<br><br>Control: 44 (27%)	Arm 1: 82%<br><br>Arm 2: 82%<br><br>Arm 3: 80%<br><br>Control: 81%	NR	Arm 1: 94%<br><br>Arm 2: 95%<br><br>Arm 3: 91%<br><br>Control: 95%
Kullgren, 2014[12]<br><br>Stage 2	Arm 1: 61.8±7.6<br><br>Arm 2: 60.7±7.4<br><br>Arm 3: 61.1±7.4	Arm 1: 211 (99%)<br><br>Arm 2: 209 (100%)	Arm 1: 44 (21%)<br><br>Arm 2: 48 (23%)<br><br>Arm 3: 43 (25%)	81%<br><br>81%<br><br>81%	NR	Arm 1: 85%<br><br>Arm 2: 87%

© 2021 Facciorusso A et al. *JAMA Network Open.*

	Control: 61.3±7.4	Arm 3: 176 (100%)<br><br>Control: 238 (100%)	Control: 53 (23%)	81%		Arm 3: 89%<br><br>Control: 91%
Mehta, 2017[30]	50-64 years	Arm 1: 198 (25.7%)<br><br>Arm 2: 186 (24.9%)<br><br>Control: 197 (26.3%)	Arm 1: 438 (58.6%)<br><br>Arm 2: 451 (60%)<br><br>Control: 463 (61.8%)	NR	NR	Only a part
Mehta, 2019[28]	Arm 1: 56 (52-63)<br><br>Arm 2: 58 (52-63)<br><br>Arm 3: 56 (52-62)<br><br>Control: 56 (52-61)	Arm 1: 101 (45.1%)<br><br>Arm 2: 101 (45.1%)<br><br>Arm 3: 94 (41.6%)<br><br>Control: 97 (43.5%)	Arm 1: 185 (82.6%)<br><br>Arm 2: 177 (79%)<br><br>Arm 3: 180 (79.6%)<br><br>Control: 170 (76.2%)	NR	Arm 1: 52 (23.2%)<br><br>Arm 2: 44 (19.6%)<br><br>Arm 3: 45 (19.9%)<br><br>Control: 47 (21.1%)	100%
Mehta, 2020 [13]	NR	Intervention: 231 (23.7%)<br><br>Control: 223 (22.6%)	NR	NR	0%	Only a part
Mehta, 2020[29]	Intervention: 57.1±5.6<br><br>Control: 56.9±5.4	Intervention: 54 (38.3%)<br><br>Control: 50 (35.7%)	Intevention: 136 (96.5%)<br><br>Control: 134 (95.7%)	NR	Intervention: 78 (55.3%)<br><br>Control: 87 (62.1%)	100%
Slater, 2018[31]	Intervention: 58.3±6.1<br><br>Control: 58.4±6.1	Intervention: 26240 (55.6%)<br><br>Control: 26225 (55.7%)	Intervention: 14528 (30.9%)<br><br>Control: 14568 (31.1%)	Intervention: 39663 (85.7%)<br><br>Control: 39618 (85.7%)	100%	100%

[Abbreviations: CRC, Colorectal Cancer; NR-Not reported]

© 2021 Facciorusso A et al. *JAMA Network Open.*

eTable 2. Risk of Bias Assessment in Individual RCTs of Financial Incentives, Using the Joanna Briggs Institute Critical Appraisal Tool

Question	Green 2019	Gupta 2016	Kullgren 2014	Mehta 2017	Mehta 2019	Mehta 2020	Mehta 2020 (II)	Nisa 2019	Slater 2019
1. Was true randomization used for assignment of participants to treatment groups?	Yes	Yes	Yes	`	Yes	Yes	Yes	Yes	Yes
2. Was allocation to treatment groups concealed?	Yes	Yes	Yes	Yes	Yes	Yes	Yes	Yes	Yes
3. Were treatment groups similar at the baseline?	Yes	Yes	Yes	Yes	Yes	Yes	Yes	Yes	Yes
4. Were participants blind to treatment assignment?	No	No	No	No	No	No	No	No	No
5. Were those delivering treatment blind to treatment assignment?	Yes	Yes	Yes	Yes	Yes	Yes	Yes	Yes	Yes
6. Were outcomes assessors blind to treatment assignment?	Yes	Yes	Yes	Yes	Yes	Yes	Yes	Yes	Yes
7. Were treatment groups treated identically other than the intervention of interest?	Yes	Yes	Yes	No	Yes	No	Yes	Yes	Yes
8. Was follow up complete and if not, were differences between groups in terms of their follow up adequately described and analyzed?	Yes	Yes	No	No	Yes	No	No	No	No
9. Were participants analyzed in the groups to which they were randomized?	Yes	Yes	Yes	Yes	Yes	Yes	Yes	Yes	Yes
10. Were outcomes measured in the same way for treatment groups?	Yes	Yes	Yes	Yes	Yes	Yes	Yes	Yes	Yes
11. Were outcomes measured in a reliable way?	Yes	Yes	Yes	Yes	Yes	Yes	Yes	Yes	Yes
12. Was appropriate statistical analysis used?	Yes	Yes	Yes	Yes	Yes	Yes	Yes	Yes	Yes
13. Was the trial design appropriate, and any deviations from the standard RCT design (individual randomization, parallel groups) accounted for in the conduct and analysis of the trial?	Yes	Yes	Yes	Yes	Yes	Yes	Yes	Yes	Yes

© 2021 Facciorusso A et al. *JAMA Network Open.*
